# A study on the factors influencing the intention to receive booster shots of the COVID-19 vaccine in China based on the information frame effect

**DOI:** 10.3389/fpubh.2024.1258188

**Published:** 2024-02-20

**Authors:** Qizhen Zhu, Yunyun Gao, Qingyuan Hu, DeHua Hu, Xusheng Wu

**Affiliations:** ^1^Department of Biomedical Informatics, School of Life Sciences, Central South University, Changsha, China; ^2^School of Medical Information Engineering, Jining Medical University, Rizhao, China; ^3^Third Xiangya Hospital, Central South University, Changsha, China; ^4^Shenzhen Health Development Research and Data Management Center, Shenzhen, Guangdong, China

**Keywords:** COVID-19 vaccine booster shot, information framing, framing effect, risk disclosure, perceived vaccine potency

## Abstract

**Introduction:**

In the response to and prevention and control of the Novel coronavirus pneumonia, the COVID-19 vaccine does not provide lifelong immunity, and it is therefore important to increase the rate of booster shots of the COVID-19 vaccine. In the field of information health science, research has found that information frames have an impact in changing individual attitudes and health behaviors.

**Objective:**

This study focuses on the effects of different influencing factors on the public’s willingness to receive the booster shots of the COVID-19 vaccine under two information frameworks.

**Methods:**

An online questionnaire was conducted to explore the effects of demographic characteristics, personal awareness, social relationships, risk disclosure, perceived booster vaccination protection rate, and duration of protection under the assumption of an information framework. T test and one-way analysis were used to testing the effect of variables.

**Results:**

(1) The persuasion effect under the gain frame is higher than that under the loss frame (*B* = 0.863 vs. *B* = 0.746); (2) There was no significant difference in subjects’ intention of booster vaccination in terms of gender, age, income, occupation, educational background and place of residence. Whether family members received booster vaccination was strongly correlated with their intention of vaccination under the loss framework (*p* = 0.017, *M* = 4.63, SD = 0.664). (3) The higher the understanding of COVID-19, the higher the degree of compliance with the government’s COVID-19 prevention and control measures, and the higher the willingness to strengthen vaccination; (4) Risk disclosure has a significant impact on people’s willingness to receive COVID-19 booster shots (*M* = 2.48, under the loss framework; *M* = 2.44, under the gain framework); (5) Vaccine protection rate and duration of protection have an impact on people’s willingness to vaccinate. Increased willingness to vaccinate when the protection rate of booster vaccine approaches 90% (*M* = 4.76, under the loss framework; *M* = 4.68, under the gain framework). When the vaccine protection period is 2 years, people are more willing to receive a booster vaccine; and the willingness to receive a booster shot is stronger under the loss framework (*M* = 4.60, SD = 0.721, *p* = 0.879).

**Conclusion:**

The impact of the information framework on COVID-19 vaccination intentions is different, and the disclosure of relevant health information should focus on the impact of the information framework and content on the public’s behavior toward strengthening vaccination. Therefore, in the face of public health emergencies, public health departments, healthcare institutions, and other sectors can consider adopting the Gainful Information Framework tool to disseminate health information to achieve better persuasion and promote public health behavior change enhancing public health awareness, and promoting universal vaccination.

## 1 Introduction

Novel coronavirus pneumonia (COVID-19), named “2019 coronavirus disease” by the World Health Organization, refers to pneumonia caused by the new 2019 coronavirus (severe acute respiratory syndrome coronavirus 2, SARS-Co V-2) infection causing pneumonia (1, 2). China Centers for Disease Control and Prevention (CDC) suggests that the new coronavirus variant currently circulating globally is still highly infectious (3). To cope with and prevent and control new coronavirus pneumonia, experts have developed the COVID-19 vaccine, which is a vaccine against the new coronavirus. However, the COVID-19 vaccine does not provide lifelong immunity, and the immune function decreases significantly after 6 months of vaccination, while the antibody level increases significantly after 6 months of the second dose of vaccine and then the booster shot, which increases more than 10 times the original level, and remains at a relatively high level after another 6 months of vaccination. And despite the fact that COVID-19 vaccination has been underway for an extended period, over 50% of the global population remains unvaccinated (4). On 13 December 2022, the Comprehensive Group of the Joint Prevention and Control Mechanism of the State Council issued the Implementation Plan for the Second Dose of Booster Immunization with the New Coronavirus Vaccine, which called for the active implementation of booster vaccination with the New Coronavirus Vaccine in China. How to better appeal to the public to receive booster shots and increase the public’s willingness to be vaccinated have become hot topics (5).

In the field of information health science, some research has found that information frames have an impact on changing individual attitudes and health behaviors. The concept of “framing effects” was first introduced by Kahneman and Tversky with the help of the Asian disease problem, where the risk preferences of individuals often depend on the way the problem is framed (6). Rothman et al. (7) suggest, based on an extensive review and evidence from previous studies, that the loss frame in disease detection behavior is a good indicator of the risk of disease. Proposed that the loss framework is more persuasive in disease detection behaviors, while the gain framework is more persuasive in disease prevention behaviors. Lee et al. (8) demonstrated through six experiments that the gain framework is more persuasive when information is dedicated to promoting behavior, and the loss framework is more persuasive when information is designed to prevent a phenomenon. These studies mentioned that information framing can have an impact on the public’s willingness and that the effects of gain and loss framing are different.

The proposed information framework provides a new theoretical perspective for the study of information behavior and a new research idea for exploring the role of information in influencing behavioral decisions. It was found that the gain frame has a stronger persuasive effect than the loss frame in prevention-biased domains such as using sunscreen to prevent skin cancer (9, 10) and quitting smoking (11–13). In contrast, vaccination is a disease prevention behavior in which the gain frame has a stronger persuasive effect than the loss frame, i.e., the gain frame has a stronger effect on people’s willingness to vaccinate (14). So, to explore the impact of information frameworks on the public’s willingness to receive COVID-19 vaccine booster shots and to understand the key factors influencing the willingness to receive COVID-19 vaccine booster shots, this paper will explore the impact of demographic characteristics, personal awareness, social relationships, risk disclosure, perceived vaccine efficacy, and duration of protection on the willingness to receive COVID-19 vaccine booster shots under different information frameworks.

## 2 Materials and methods

### 2.1 Investigation method

This study investigated the effect of information frames on people’s willingness to receive COVID-19 vaccine booster shots and was conducted by the guidelines of the Declaration of Helsinki. Two different types of questionnaires (loss frame (Paper A) and gain frame (Paper B)) were distributed through an online questionnaire platform, and links to the questionnaires were distributed to participants through social software such as WeChat and QQ. The questionnaire stated “If you have already filled out the volume A, please do not fill out the volume B” to exclude the possibility of cross-filling.

The questionnaire first introduced the current status and progress of epidemic prevention and control in China and the concept and role of booster shots (booster immunization). The questionnaire consisted of three main parts, firstly, basic information about the subject, such as gender, age, occupation, place of residence, education level, income, and the subject’s and subject’s family members’ information about the COVID-19 vaccination booster shot and knowledge about COVID-19 and compliance with government COVID-19 prevention and control measures. In the second part, the questionnaire provided four stimulus messages designed according to the characteristics of gain and loss frames, and subjects continued to respond to the intention to receive the COVID-19 vaccine booster after carefully reading the stimulus messages. In the last part, subjects continued to read and respond to questions about COVID-19 vaccine booster effectiveness, side effects, duration of protection, and family member vaccination rates.

All participants gave informed consent and volunteered to participate in this study. Individuals with mental illness or cognitive impairment and those who refused to participate in the study were excluded (When investigating the basic information of the subject, there are relevant questions asking whether he/she has a mental illness or not) (15).

We distributed questionnaires nationwide from January 2023 until June 2023.A combination of random distribution and snowball sampling for online survey distribution to expand the sample size. 594 questionnaires were sent out, 540 valid questionnaires were obtained, after removing invalid questionnaires, and the response rate was 90.45%. Including 273 questionnaires based on the gain framework and 267 questionnaires based on the loss framework.The collected data were analyzed by SPSS 21.0, and the Cronbach’s alpha values of the two groups were 0.769 and 0.753, both greater than 0.7, which proved that the questionnaire had good reliability, and the KMO values were 0.751 and 0.765, both greater than 0.6, which proved that the questionnaire had good validity and could be analyzed formally.

### 2.2 Variable measurement and statistical analysis

This study involved measures of intention to vaccinate against COVID-19 and in various framing scenarios, all of which used the Likert 5 scale, the higher the value, the higher the effect of information framing, and the higher the intention to vaccinate against COVID-19. All measures of relevant factors were adapted from existing studies and finalized as follows.

1. The vaccination intention for COVID-19 was adapted from Fishbein and Ajzen (16)“What is your intention for COVID-19 vaccine booster vaccination?”; “If COVID-19 booster vaccination is available for a fee, how likely are you to receive it?”; “If there is a waiting list for the COVID-19 booster vaccination, how likely are you to get the vaccination?”
2. Intention to get vaccinated was measured in four specific combinations with different frameworks of information, including vaccine effectiveness, safety, duration of protection, and family member vaccination rates, such as “If the COVID-19 booster is 60% effective, how likely are you to get the vaccine booster?; “If the COVID-19 booster vaccine causes side effects such as swelling and pain at the vaccination site, itching, generalized fever, and malaise, how likely is it that you will receive the booster?”; “If the duration of protection for the COVID-19 vaccine booster is 2 years (24 months), how likely is it that you will receive the vaccination?” What is the likelihood that you will receive the booster vaccine if 90% of the family members around you receive it?” etc.;
3. Based on the existing research base, this study involved nine control variables: gender, age, occupation, place of residence, literacy, income, whether family members received COVID-19 vaccine booster shots, knowledge of COVID-19, and compliance with government COVID-19 prevention and control measures.

Two variables (gender and place of residence) were subjected to an independent samples t-test and four variables (age, occupation, income, and education) were subjected to one-way analysis of variance (ANOVA).Finally, we conducted regression analyses between different information frames and the public’s willingness to receive the COVID-19 vaccination booster.

### 2.3 Information framing

There are two types of information frames: the benefits of taking action - i.e., gain-frame information (e.g., getting a New Crown booster vaccination is effective in preventing COVID-19 infection), and the costs of refusing to take action - i.e., loss-frame information (e.g., by not receive a New Crown vaccine booster, you are likely to become infected with COVID-19). The loss-framed information is more compelling in disease detection behaviors, while the gain-framed information is more compelling in disease prevention behaviors (17).

A study showed that gain-frame messages were more persuasive when the message was intended to promote a behavior, whereas loss-frame messages were more persuasive when the message was intended to prevent a phenomenon (14). It has been found that gain-frame messages are more effective than their loss-frame counterparts in areas that favor prevention. Whereas vaccination is a disease prevention behavior, it is known that gain-frame messages are more persuasive than loss-frame messages in the preventive behavior of vaccination, i.e., gain-frame messages have a stronger effect on people’s willingness to receive booster shots (18).

However, when the degree of risk of taking, and refusing a behavior varies, people’s willingness is affected as a result: the persuasive effect of gain-framing information is stronger when the risk of behavioral consequences is low, and the effect of loss-framing information is stronger when the risk of behavioral consequences is high (19). Compared with general preventive behaviors, the risk of booster vaccination may be higher and may cause a series of reactions such as swelling and pain at the vaccination site, itching, generalized fever and malaise, cerebral thrombosis and stroke, and even life-threatening, i.e., booster vaccination is a high-risk behavior. In summary, booster vaccination is both a preventive and a high-risk behavior. Therefore, which framing message has a stronger effect on the intention to receive booster vaccine needs further study.

All information covered in this study was designed based on the characteristics of the gain and loss frames from the literature review, resident interviews, and expert consultations. In addition, the number of words between the two frames was similar in order to avoid the influence of the amount of information. The stimulus information is shown in Table 1.

**Table 1:** Stimulation information used in the questionnaire.

Information	Loss-framed messages	Gain-framed messages
1	Wang Huaqing, chief expert in immunization planning at the CDC, said, “After a period of vaccination with the new coronavirus, some people experience a decline in immunity to the new coronavirus, and there is a weakened protective effect.” Without the booster vaccination, there is no way to get a large number of more durable neutralizing antibodies to “rebound” from this decline in immunity and produce a better protective effect.	Academician Zhong Nanshan said that with inactivated vaccines or mRNA vaccines, there is a significant decrease in immune function 6 months after vaccination. Six months after the second dose of the vaccine and then the booster shot, there was a significant increase in antibody levels, which increased by more than 10 times the original level. The level of antibodies was maintained at a relatively high level for another 6 months after vaccination, which means that the booster shot produced more durable antibodies. This also means that the booster shot is more effective. In addition, the booster vaccination is also very safe.
2	At present, some mutant strains may have some “immune escape” phenomenon, simply put, after the mutation of the new coronavirus, the combined effect of antibodies produced by vaccination on the virus will be weakened, and without the booster shot only rely on the previously vaccinated new coronavirus to deal with the mutant strains may not have sufficient targeting ability to fight the mutant strains.	Related studies have shown that in addition to enabling an increase in antibodies, the booster shot also has a broader antibody spectrum, enabling effective targeting of a wider variety of viral strains, meaning that it produces better targeting of variant strains and that different New Crown vaccine booster immunization strategies can increase the body’s ability to neutralize Omicron strains by a factor of 8–15.
3	Recent data from the Hong Kong epidemic show that not receiving a booster shot increases the disease and death rate substantially. The morbidity and mortality rate was 0.03% for three vaccinations, 0.14% for two vaccinations, 0.96% for one vaccination, and 3.2% for no vaccination.	Zhang Wenhong, director of the National Medical Center for Infectious Diseases and head of the Shanghai New Crown Pneumonia Medical Treatment Expert Group, said, “The third dose of the vaccine is very protective against severe illness and death, providing more than 95% protection against severe illness and death.
4	Zhang Wenhong, director of the National Medical Center for Infectious Diseases and head of the Shanghai New Coronary Pneumonia Medical Treatment Expert Group, said that people will eventually be unable to take off their masks if the third immunization is not reinforced.	Academician Li Lanjuan said that at this time when the epidemic is spreading worldwide, it is hoped that those who have already completed the full course of vaccination against Neocon and are eligible for the vaccine should get a “booster” as soon as possible so that they can build an immune barrier against Neocon and improve their immunity.

### 2.4 Personal cognition and social relationships

Personal cognition is a mental function that involves the storage, selection, organization, and action planning of information. In the field of cognitive epidemiology, it is believed that there is an association between individual cognition and mortality, disease, and health. In our study, personal cognition refers to people’s knowledge of COVID-19 and their awareness of compliance with government prevention and control measures, which may be associated with willingness to receive the COVID-19 booster vaccine. Also, a study showed that whether an individual’s loved ones, family members, or public figures they trust and respect receive booster shots influences that person’s willingness to receive booster vaccines (20). That is, booster vaccination of family members affects an individual’s willingness to receive a booster vaccine.

### 2.5 Risk disclosure

In the field of health communication, risk disclosure refers to the decision to accept or reject a medical intervention or medication by providing patients with important risk information (21). A Study of Intention to Vaccinate Against Seasonal Influenza Virus Shows that a Communication model framed by risk disclosure information leads to lower public intention to receive influenza vaccine (22). Therefore, disclosure of information on the risks of the COVID-19 vaccine booster may affect the public’s willingness to receive the vaccine.

### 2.6 Perceived vaccine effectiveness and duration of protection

Perceived vaccine effectiveness refers to the “effectiveness of the vaccine” and is the main consideration for individuals to receive vaccines (23). A study of vaccination behavior showed that information framing and vaccination intentions were moderated by perceived vaccine effectiveness (24), suggesting that target framing information would increase people’s belief in vaccine effectiveness, thereby increasing their intentions to receive a booster vaccine. Duration of immunity also has a positive impact on the public’s willingness to be vaccinated.

## 3 Results

### 3.1 Influence of demographic characteristics on intention to receive booster vaccinations

#### 3.1.1 Effect of demographic characteristics on vaccination intentions based on a gaining framework

According to Table 2, there was no significant difference between male and female subjects in the case of receiving the gaining frame information by statistical analysis, and there was no significant difference between male and female subjects in terms of elevated intention to receive booster vaccination. Gender, place of residence, average monthly income occupation, and education level did not reach significant levels for booster vaccination. In addition, the willingness to receive booster vaccination was greater in the group aged 50–59 years than in other age groups; the willingness to receive booster vaccination was greater in the group with an average monthly income of RMB 3001–5,000 than in other income levels.

**Table 2:** Effect of demographic characteristics on booster vaccination intention based on a gaining framework (*N* = 267).

Variable	Category	*N* (%)	*M*	SD	*F*	*p*
Gender	Male	124(46.44%)	4.73	0.616	0.479	0.489
	Female	143(53.56%)	4.74	0.527		
Residence	City	186(69.66%)	4.72	0.576	1.108	0.294
	Rural	81(30.34%)	4.77	0.554		
Age	<18	4(1.5%)	4.00	1.414	2.682	0.032
	18–29	152(56.93%)	4.76	0.524		
	30–49	94(35.21%)	4.68	0.608		
	50–59	11(4.12%)	5.00	0.000		
	≥60	6(2.25%)	4.83	0.408		
Revenue (Yuan)	≤3000元	142(53.18%)	4.74	0.555	1.520	0.197
	3,001 ~ 5,000元	25(9.36%)	4.92	0.400		
	5,001 ~ 10000元	56(20.97%)	4.73	0.556		
	10,001 ~ 20000元	31(11.61%)	4.55	0.723		
	>20000元	13(4.87%)	4.77	0.599		
Occupation	Student	138(51.69%)	4.75	0.551	1.717	0.131
	Civil servant	8(3%)	4.38	1.061		
	Employees of enterprises/ institutions	90(33.71%)	4.74	0.531		
	Self-employed/Freelancer	13(4.87%)	4.85	0.555		
	Farmers	11(4.12%)	4.82	0.405		
	Others	7(2.62%)	4.29	0.756		
Education	Junior college and below	36(13.48%)	4.75	0.649	0.152	0.859
	Undergraduate	182(68.16%)	4.74	0.570		
	Master’s degree and above	49(18.35%)	4.69	0.508		

#### 3.1.2 Effect of demographic characteristics on vaccination intentions based on a loss framework

According to the following Table 3, the willingness to vaccinate was statistically higher in men than in women in the case of loss of frame information, and male subjects had an increased willingness to vaccinate when receiving loss of frame information, which may be related to the higher risk of COVID-19 complications and death in men (25). Place of residence, age, mean monthly income, occupation, and education did not reach significant levels for booster vaccination. In addition, booster vaccination intentions were greater in groups younger than 18 years than in all other age groups; booster vaccination intentions were greater in groups with an average monthly income of 3,001–5,000 yuan than in groups with other income levels.

**Table 3:** Effect of demographic characteristics on booster vaccination intention based on a loss framework (*N* = 273).

Variable	Category	*N* (%)	*M*	SD	*F*	*p*
Gender	Male	136(49.82%)	4.68	0.540	10.819	0.001
	Female	137(50.18%)	4.53	0.805		
Residence	City	198(72.53%)	4.58	0.699	2.278	0.132
	Rural	75(27.47%)	4.69	0.657		
Age	<18	8(2.93%)	4.75	0.463	0.309	0.872
	18–29	135(49.45%)	4.63	0.710		
	30–49	113(41.39%)	4.59	0.703		
	50–59	10(3.66%)	4.50	0.527		
	≥60	7(2.56%)	4.43	0.535		
Revenue (Yuan)	≤3000元	122(44.69%)	4.67	0.581	1.189	0.316
	3,001 ~ 5,000元	34(12.45%)	4.71	0.676		
	5,001 ~ 10000元	60(21.98%)	4.57	0.722		
	10,001 ~ 20000元	40(14.65%)	4.45	0.749		
	>20000元	17(6.23%)	4.47	1.068		
Occupation	Student	114(41.76%)	4.67	0.605	0.848	0.517
	Civil servant	10(3.66%)	4.70	0.483		
	Employees of enterprises/institutions	114(41.76%)	4.51	0.823		
	Self-employed/Freelancer	16(5.86%)	4.69	0.479		
	Farmers	11(4.03%)	4.73	0.467		
	Others	8(2.93%)	4.75	0.463		
Education	Junior college and below	53(19.41%)	4.70	0.575	2.815	0.062
	Undergraduate	163(59.71%)	4.64	0.682		
	Master’s degree and above	57(20.88%)	4.42	0.778		

### 3.2 Influence of personal cognition and social relationships on intention to receive booster vaccinations

#### 3.2.1 Influence of individual cognition on intention to receive booster vaccinations

According to Table 4, by one-way analysis of variance (ANOVA), we found that knowledge of COVID-19 (*p* = 0.000 < 0.05) and compliance with government COVID-19 control measures (*p* = 0.002 < 0.05) were significantly associated with intention to receive booster vaccination in a gain framework. Knowledge of COVID-19 (*p* = 0.001 < 0.05) and compliance with government COVID-19 control measures (*p* < 0.001 < 0.05) were also significant in the loss frame. This implies that higher knowledge of COVID-19 is associated with greater compliance with government COVID-19 control measures and higher willingness to receive booster vaccinations.

**Table 4:** Influence of individual awareness on intention to receive booster vaccinations.

Variable	Information framing	Category	*N* (%)	*M*	SD	*F*	*p*
Level of knowledge of novel coronaviruses	Loss	Very uninformed	2(0.73%)	3.00	2.828	4.587	0.001
		Relatively unaware	12(4.4%)	4.42	0.669		
		Uncertain	42(15.38%)	4.62	0.764		
		Comparative aware	159(58.24%)	4.57	0.689		
		Very well informed	58(21.25%)	4.81	0.395		
Degree of compliance with government COVID-19 precautionary measures		Uncertain	1(0.37%)	4.00		11.717	0.000
		Comparative aware	40(14.65%)	4.15	0.662		
		Very well informed	232(84.98%)	4.69	0.663		
Level of knowledge of novel coronaviruses	Gain	Relatively unaware	13(4.87%)	4.77	0.439	10.180	0.000
		Uncertain	37(13.86%)	4.30	0.878		
		Comparative aware	169(63.3%)	4.78	0.509		
		Very well informed	48(17.98%)	4.92	0.279		
Degree of compliance with government COVID-19 precautionary measures		Comparative non-compliance	1(0.37%)	4.00	.	5.018	0.002
		Uncertain	1(0.37%)	5.00	.		
		Comparative compliance	18(6.74%)	4.28	0.958		
		Very compliance	247(92.51%)	4.77	0.517		

The values of “very unaware” for the gain frame of “Knowledge of COVID-19” and “very non-compliant, relatively non-compliant” and “very non-compliant” for the loss frame of “Compliance with government COVID-19 precautions” in Table 3 were not selected by anyone (*N* = 0) and therefore no statistical results are shown.

The value of *p* of 000 occurs because the probability of significance of the statistical test is extremely small, and its number is all zeroes before the retained valid decimal places, so it is fixed to show 0.000.

#### 3.2.2 Effect of social relationships on intention to receive booster vaccinations

According to the data in Table 5, we conducted a comparative analysis of changes in the public’s willingness to receive COVID-19 vaccine booster across different information frames at 30 per cent, 60 per cent and 90 per cent vaccination rates for family members.

**Table 5:** Effect of booster vaccination rate among acquaintances on intention to receive booster vaccination.

Variable	Information framing	*M*	SD	*T*	*p*
30%	Loss	3.84	1.025	61.842	0.000
	Gain	3.83	1.030	60.737	0.000
60%	Loss	4.28	0.789	89.683	0.000
	Gain	4.25	0.837	83.024	0.000
90%	Loss	4.70	0.624	124.428	0.000
	Gain	4.61	0.734	102.573	0.000

Vaccination rates of acquaintances were significantly correlated with vaccination intentions under all information framework assumptions (*p* < 0.001), and after receiving information about vaccination rates of acquaintances, subjects’ vaccination intentions increased with the increase in vaccination rates of acquaintances. With 30% of the acquaintances around them vaccinated, people are holding a wait-and-see attitude.

As seen in Table 6 there was a significant difference between the change in vaccination intention by whether family members were vaccinated or not in the loss framework (*p* = 0.017 < 0.05). When family members were vaccinated with the new crown vaccine (mean = 4.63, standard deviation = 0.664), their willingness to vaccinate was much higher than those without family members vaccinated (mean = 4.10, standard deviation = 1.101). With the gain frame information, there was no significant difference between whether family members/friends were vaccinated or not on subjects’ willingness to be vaccinated (*p* = 0.114 > 0.05), which did not reach statistical significance.

**Table 6:** Effect of family members’ vaccination on intention to receive booster shots.

Variable	Information framing	Category	*N*(%)	*M*	SD	*F*	*p*
Vaccination status of booster shots for family members	Loss	Vaccinated	263(96.34%)	4.63	0.664	5.745	0.017
		Unvaccinated	10(3.66%)	4.10	1.101		
	Gain	Vaccinated	253(94.76%)	4.75	0.556	2.515	0.114
		Unvaccinated	14(5.24%)	4.50	0.760		

### 3.3 Impact of information framework on intention to receive booster vaccination

The information frames involved in this study were divided into loss-frame and gain-frame groups. As shown in Table 7, the mean intention to receive COVID-19 vaccine was lower in the loss-frame group (mean = 4.62, standard deviation = 0.595; mean = 4.11, standard deviation = 0.959) than in the gain-frame group (mean = 4.72, standard deviation = 0.550; mean = 4.23, standard deviation = 0.960). The information frame was significantly associated with the intention to vaccinate with COVID-19 (*p* < 0.001). However, the comparison between the two groups was not significant.

**Table 7:** Linear regression analysis of vaccination intention for booster shots in an information framework.

Variable	Information framing	*M*	SD	*R* ^2^	*B*	*T*	*p*
The intention of booster vaccination	Loss	4.62	0.595	0.557	0.746	128.210	0.000
	Gain	4.72	0.550	0.745	0.863	140.175	0.000
Willingness to charge for vaccination booster shots	Loss	4.11	0.959	0.242	0.492	70.780	0.000
	Gain	4.23	0.960	0.254	0.504	71.985	0.000

Further analysis of the linear regression analysis revealed that for booster vaccination intention, We conducted regression analyses using different information frames used as independent variables, public willingness to receive the COVID-19 vaccine booster vaccination as well as public willingness to receive the COVID-19 booster vaccine in a fee-paying situation as dependent variables, the absolute value of the standardized beta coefficient under the loss framework was smaller than under the gain framework (B = 0.746 < B = 0.863, *p* < 0.001), suggesting that the gain framework has a greater effect on intention to receive COVID-19 vaccination than the loss framework. With an emphasis on booster vaccination charges, the willingness to receive booster vaccination decreases in both frameworks. The introduction of a free booster vaccination policy in China has, to some extent, increased the coverage of booster vaccination. In contrast, the absolute values of the standardized Beta coefficients in the loss frame were smaller than those in the gain frame (B = 0.492 < B = 0.504, *p* < 0.001) for whether or not they intended to charge for booster vaccination, indicating that the gain frame had a larger effect on intention to charge for COVID-19 vaccine booster vaccination than the loss frame and that the standardized Beta coefficients between the loss and gain frames The comparison of absolute values was not particularly significant.

### 3.4 Impact of risk disclosure on intention to receive booster vaccinations

According to the data in the following Table 8, compares differences in public willingness to be inoculated under different information frameworks when faced with disclosed risk factors, the intention of the subjects changed significantly after receiving information about the side effects of the COVID-19 vaccine. People may opt for booster vaccination in the loss frame (mean = 3.48, standard deviation = 1.122, *p* < 0.001) but tend to wait and see in the gain frame (mean = 3.34, standard deviation = 1.163, *p* < 0.001) if the side effects of the vaccine are swelling and pain, itching, generalized fever, and malaise. In addition, when vaccination may lead to cerebral thrombosis and stroke, the effect of the two information frames on people’s willingness to receive booster vaccination was similar (mean = 2.22, standard deviation = 1.190, *p* < 0.001; mean = 2.23, standard deviation = 1.191, *p* < 0.001) and people’s willingness to receive booster vaccination compared to side effects were “swelling and pain, itching, generalized fever, and weakness.” And if vaccination affects life safety and has a risk of death, people mostly tend to refuse (mean = 1.75, standard deviation = 1.221, *p* < 0.001; mean = 1.77, standard deviation = 1.265, *p* < 0.001). Different levels of risk disclosure led to a significant decrease in vaccination intention compared to no disclosure of these risks (mean = 4.61, standard deviation = 0.689, *p* < 0.001; mean = 4.73, standard deviation = 0.569, *p* < 0.001).

**Table 8:** Impact of risk disclosure on intention to receive booster vaccinations.

Vaccine side effect information	Information framing	*M*	SD	*T*	*p*
Swelling and pain, itching, generalized fever, weakness	Loss	3.48	1.122	51.317	0.000
	Gain	3.34	1.163	46.894	0.000
Cerebral thrombosis, stroke	Loss	2.22	1.190	30.864	0.000
	Gain	2.23	1.191	30.584	0.000
Death	Loss	1.75	1.221	23.643	0.000
	Gain	1.77	1.265	22.836	0.000
Not disclosed yet	Loss	4.61	0.689	110.531	0.000
	Gain	4.73	0.569	135.950	0.000

### 3.5 Effect of perceived booster vaccine protection rate on vaccination intention

Based on the data in Table 9, explored the impact of the public’s perceived protection rate of booster vaccines on vaccination intentions under different information frameworks, the perceived booster vaccine protection rate was significantly associated with intention to receive the booster vaccine under all information framework assumptions (*p* < 0.001). After receiving information about the booster shot protection rate of the COVID-19 vaccine, subjects’ intentions decreased in the first two degrees (30, 60%) and increased in the third degree (90%). When the booster vaccine protection rate was 30%, there was a tendency to adopt a wait-and-see attitude (mean = 3.69, standard deviation = 1.112, *p* < 0.001; mean = 3.58, standard deviation = 1.181, *p* < 0.001). When the protection rate of the booster vaccine was 60%, people were likely to be willing to receive the booster vaccine (mean = 4.24, standard deviation = 0.898, *p* < 0.001; mean = 4.16, standard deviation = 0.910, *p* < 0.001). People tended to be very willing to receive the booster vaccine when the protection rate of the booster vaccine was 90% (mean = 4.76, standard deviation = 0.706, *p* < 0.001; mean = 4.68, standard deviation = 0.781, *p* < 0.001). This suggests that when the protection rate of booster vaccine approaches 90%, the promotion of vaccine protection rate can appropriately increase people’s willingness to receive booster vaccine (mean = 4.61 vs. mean = 4.76; mean = 4.73 vs. mean = 4.68).

**Table 9:** Effect of perceived booster vaccine protection rate on vaccination intention.

Variable	Information framing	*M*	SD	*T*	*p*
30%	Loss	3.69	1.112	54.878	0.000
	Gain	3.58	1.181	49.476	0.000
60%	Loss	4.24	0.898	77.946	0.000
	Gain	4.16	0.910	74.750	0.000
90%	Loss	4.76	0.706	111.465	0.000
	Gain	4.68	0.781	97.856	0.000

### 3.6 Effect of perceived duration of booster vaccine protection on vaccination intention

According to the data in Table 10, after receiving information about the duration of protection for COVID-19 vaccine booster shots, subjects’ intentions were slightly lower in the first two degrees (6 months, 1 year) and the third degree (2 years) than before receiving the hypothesis (mean = 4.60 vs. mean = 4.61; mean = 4.58 vs. mean = 4.73). When the duration of protection was 6 months, people were likely to choose wait-and-see vaccination in both the loss and gain frames (mean = 3.92, standard deviation = 0.967, *p* < 0.001; mean = 3.92, standard deviation = 1.010, *p* < 0.001); when the duration of protection was 1 year, people were likely to choose booster vaccination in both the loss and gain frames (mean = 4.29, standard deviation = 0.828, *p* < 0.001; mean = 4.29, standard deviation = 0.861, *p* < 0.001); when the duration of protection was 2 years, people were more likely to receive booster vaccine and were slightly more likely to receive a booster shot in the loss frame (mean = 4.60, standard deviation = 0.721, *p* = 0.879) than in the gain frame (mean = 4.58, standard deviation = 0.758, *p* = 0.002).

**Table 10:** Effect of perceived booster vaccine protection rate on vaccination intention.

Variable	Information framing	*M*	SD	*T*	*p*
Half year (6 months)	Loss	3.92	0.967	66.985	0.000
	Gain	3.92	1.010	63.441	0.000
One year (12 months)	Loss	4.29	0.828	85.650	0.000
	Gain	4.29	0.861	81.452	0.000
Two years (24 months)	Loss	4.60	0.721	105.401	0.000
	Gain	4.58	0.758	98.682	0.000

In summary, the minimum acceptable standard of protection for the COVID-19 vaccine is at least 1 year. People are more inclined to protect themselves and choose wait-and-see rather than vaccinate with a protection period of less than 1 year.

### 3.7 Multi-factor impact analysis under different information frameworks

From the data in Table 11, multivariate analysis Of Variance, we used a multifactorial analysis of variance to analyse the multifactorial effects on the willingness to receive a booster shot of the New Crown vaccine across different information frames, also we include gender as a control variable to exclude its confounding effect. Under the loss framework, social relationships, Level of knowledge of novel coronaviruses, and Risk Disclosure have a significant effect on willingness to receive booster shots under a multifactorial mixture of influences (*p* < 0.05), this is consistent with previous analyses. Under the gain framework, Level of knowledge of novel coronaviruses, Degree of compliance with government COVID-19 precautionary measures, and Risk Disclosure have an impact on willingness to receive booster shots (*p* < 0.05).

**Table 11:** Multifactorial effects on vaccination intention.

Variable	Information framing	DF	MS	*F*	*p*
Social relationships	Loss	1	0.2.727	8.493	0.004
	Gain	1	0.003	0.167	0.683
Level of knowledge of novel coronaviruses	Loss	4	1.996	6.216	0.000
	Gain	3	0.134	6.627	0.000
Degree of compliance with government COVID-19 precautionary measures	Loss	1	0.785	2.446	0.119
	Gain	2	0.043	2.115	0.000
Risk disclosure	Loss	4	1.432	4.460	0.002
	Gain	4	0.054	2.683	0.033

## 4 Discussion

### 4.1 Influence of demographic characteristics on willingness to vaccinate booster shoot

Previous research has extensively explored the impact of socio-demographic factors on vaccine uptake, yet there is a dearth of studies elucidating the differential effects of socio-demographic characteristics within the context of a gain-loss framework. Men exhibit a greater willingness to receive the COVID-19 booster shot in a loss framework, possibly due to the elevated risk of complications and mortality in men (26). This heightened persuadability aligns with men’s lower trust in vaccine-related rumors and greater awareness of the infectious disease risk (27, 28).Contrary to expectations, age showed no significant association with vaccination willingness in the information framework, though prior studies suggest older individuals are more inclined to vaccinate (29, 30). This discrepancy may be attributed to the study’s focus on the 18–49 age group, limiting representation of older individuals. While previous research indicates a positive link between high income and vaccination willingness (31, 32), our findings diverge within the loss framework. High-income groups (> $10,000 monthly) demonstrate lower willingness, possibly due to their cautious approach, access to information, and financial stability for protective measures. Additionally, the higher-educated group (master’s degree and above) exhibits lower vaccination willingness, likely stemming from their ability to process more information and prioritize safety and efficacy considerations. This nuanced response highlights the multifaceted influences on vaccination attitudes.

### 4.2 Impact of personal cognition on willingness to vaccinate booster shoot

This study establishes a positive correlation between higher knowledge of COVID-19 and increased willingness to vaccinate it (33–35). Alignment with government-mandated prevention measures also positively influences vaccination intent. Trust in the government, particularly in countries like Korea and China, where vaccine uptake is associated with governmental trust, plays a crucial role (34). Moreover, individuals exhibiting high trust in their country’s health system or information disseminated by public health authorities express greater willingness to receive the vaccine (36, 37).

### 4.3 Impact of social relationships on willingness to vaccinate booster shoot

The study indicates that a higher vaccination rate among close contacts positively influences individuals’ willingness to get vaccinated. About 78% of participants cited the influence of family and friends in their vaccination decision (38). Having vaccinated family or friends significantly increases vaccination intent in the loss framework, with inconsistent results in the gain framework (19).

China’s effective epidemic control may lead to complacency and reduced risk perception, potentially causing hesitancy (39). However, a loss-framed approach emphasizes the consequences of not vaccinating, highlighting continued disease spread and challenges in achieving herd immunity. This may prompt individuals to vaccinate, especially if their social circle is actively doing so.

However, a 60% acquaintance vaccination rate is still not effective in persuading people to receive the vaccine in either a loss or gain information framework, so there is some difficulty in achieving a herd immunization rate by people consciously receiving the vaccine.

Beliefs in individual and community benefits positively impact vaccination intent (40). Emphasizing communal advantages could motivate hesitant individuals to vaccinate. Targeting those with low vaccination intentions, persuading their social network, or engaging influential community figures may enhance overall vaccination intentions.

### 4.4 Impact of information framing on willingness to vaccinate booster shoot

The information frame can influence willingness to receive a booster vaccination with the COVID-19 vaccine, with the gain frame outweighing the loss frame for both intention to receive a booster vaccination and intention to charge for a booster vaccination. While the intention to receive booster vaccination generally declines when we emphasize the cost of booster vaccination for a fee. A study indicates that the provision of free COVID-19 vaccination services is crucial for low- and middle-income countries (41), a principle that holds true in China as well. It is noteworthy that the comparison of vaccination impacts between the two frames is similar when booster vaccination is charged for.

### 4.5 Risk disclosure effect of willingness to vaccinate booster shoot

Disclosure of potential side effects of booster shots for the COVID-19 vaccine significantly reduces vaccination willingness, particularly within a loss framework. This conclusion is largely consistent with the perspective of the Prospect Theory, wherein individuals choose vaccination to mitigate greater risks. However, when the act of vaccination itself entails substantial risks, exceeding the risk of disease from refusal, people tend to decline vaccination. Some studies indicate that individuals perceiving higher infection risks are more likely to accept the vaccine (42–45). This inconsistency may be related to effective epidemic control in our country, resulting in a relatively lower infection risk. Therefore, excessive transmission of risk information regarding booster shots may lead the public to perceive the risks of not vaccinating as lower than the vaccine’s side effects.

### 4.6 Effect of perceived vaccine effectiveness and duration of protection

In a study assessing the vaccination willingness of Hong Kong residents against COVID-19, a correlation was observed between perceived vaccine effectiveness and the duration of protection, a finding congruent with our own research (46). The expeditious development of the COVID-19 vaccine, influenced by the unique spread of the virus, has engendered uncertainty regarding immunization effectiveness and its duration, thereby diminishing perceived vaccine efficacy (47).

The United States Food and Drug Administration has established a minimum acceptable effectiveness threshold of 50% for the COVID-19 vaccine (48). Achieving universal immunity necessitates a high population coverage exceeding 70% (49). Notably, a study underscores that, even with an 80% vaccine effectiveness rate, a minimum vaccination coverage of 75% is indispensable for pandemic control (50). Given the prevalent low perception of the efficacy of the newly available COVID-19 vaccine, it becomes imperative to incorporate information on the vaccine’s effective protection rate in booster dose promotion efforts to enhance public willingness to undergo vaccination.

### 4.7 Multifactorial effect under different information frameworks

Multi-factor impact analysis of two different information frameworks, we found that in the loss frame, the degree of compliance with government COVID-19 precautionary measures did not have an impact on the willingness to step up vaccination. Possible reasons for this are that the public, after receiving information about the costs of refusing booster shots, will be less interested in complying with the Government’s new crown precautionary measures and will be more concerned about social relations, knowledge of novel coronaviruses and risk disclosure. On the contrary, under the benefit framework, the public receives various information related to the benefits of receiving booster shots, and the thoughts of those around them may converge, so the influence of social ties on the willingness to receive booster shots of the new crown vaccine may not be as prominent as compared with other factors under the influence of the multiple factors.

## 5 Conclusion

This study explored the factors influencing the intention to receive COVID-19 vaccine booster shots based on information framing. Firstly, concerning sociodemographic characteristics, there is a strong correlation between household members receiving booster vaccine shots and individual willingness to receive them under the gain framework. Other factors such as gender, age, income, occupation, educational background, and place of residence did not show significant differences. Secondly, a higher level of knowledge about COVID-19 and greater adherence to government COVID-19 prevention and control measures were associated with a higher willingness to receive booster vaccination. Additionally, the persuasive effect of the gain framework was found to be higher than that of the loss framework in the context of COVID-19 vaccine booster shots. Finally, risk disclosure significantly influenced individuals’ willingness to receive COVID-19 vaccine booster shots.

Based on the findings above, we propose the following recommendations to increase the COVID-19 vaccine booster shot coverage: Firstly, public health departments and healthcare institutions should raise awareness about COVID-19 among the public, and the government should enhance the dissemination of preventive measures. Secondly, public health departments, healthcare institutions, and other relevant sectors should focus on utilizing the gain framework to promote health behaviors. Thirdly, withholding risk disclosure information during information dissemination can effectively enhance individuals’ willingness to receive booster vaccine shots. Therefore, when administering booster shots, medical information of residents should be kept as confidential as possible, accompanied by privacy protection statements, to address public concerns and thereby expand booster vaccination coverage.

## 6 Limitations and directions for future research

The results of this study provide theoretical and practical insights in the area of promoting public health behaviors. The gain framework had a greater persuasive effect than the loss framework in terms of willingness to vaccinate for new crowns. The concept that risk disclosure has a large negative impact on vaccination intention may be a meaningful predictor for testing vaccine promotion information strategies. However, shortcomings remain in the study:

On the one hand, this study used a convenience sampling method, and despite the randomness of the sample, most of the participants were a young and middle-aged group, the age distribution of the sample is more restricted, and the information framing effect on the willingness to vaccinate against COVID-19 vaccine in older age groups needs to be further studied.

On the other hand, the study mainly explored the effects of various factors on the public’s willingness to receive the New Corona booster vaccination under different information frameworks, however, the factors affecting the public’s willingness to vaccinate are multifaceted, and only some of them have been selected for study in this paper. In future studies, other factors can be explored to influence vaccination intentions, such as the influence of third-party organizations’ interventions. Also, future research could compare a range of behavioral changes in the public under different information frameworks to examine the relationships and underlying mechanisms between these behaviors.

There is still much room for exploring the application of information frameworks in the health field, and there are various forms of research methods to broaden the research and application of information frameworks in the health field, in addition to the gain and loss frameworks involved in this study.

## Data availability statement

The original contributions presented in the study are included in the article/supplementary material, further inquiries can be directed to the corresponding authors.

## Ethics statement

The studies involving human participants were reviewed and approved by the Institutional Review Board of the College of Life Sciences, Central South University (Reference No. 2022-1-45). The participants provided their written informed consent to participate in this study. Written informed consent was obtained from the individual(s) for the publication of any potentially identifiable data included in this article.

## Author contributions

QZ: Data curation, Formal analysis, Investigation, Methodology, Writing – original draft. YG: Data curation, Formal analysis, Investigation, Writing – original draft. QH: Data curation, Investigation, Writing – original draft. DH: Conceptualization, Supervision, Validation, Visualization, Writing – review & editing. XW: Data curation, Project administration, Validation, Visualization, Writing – review & editing.
